# Supplementary material for: JUMPlion improves quantitative DIA proteomics through ion-level recovery of missing values
Source: bioRxiv. 2026 May 1:2026.04.28.720212. Preprint. [Version 1] doi: 10.64898/2026.04.28.720212 (PMC13142488; doi:10.64898/2026.04.28.720212)
Supplement: 1 [file NIHPP2026.04.28.720212v1-supplement-1.pdf]

bioRxiv preprint doi: https://doi.org/10.64898/2026.04.28.720212; this version posted May 1, 2026. The copyright holder for this preprint (which was not certified by peer review) is the author/funder, who has granted bioRxiv a license to display the preprint in perpetuity. It is made available under aCC-BY 4.0 International license.

Supplementary Figures

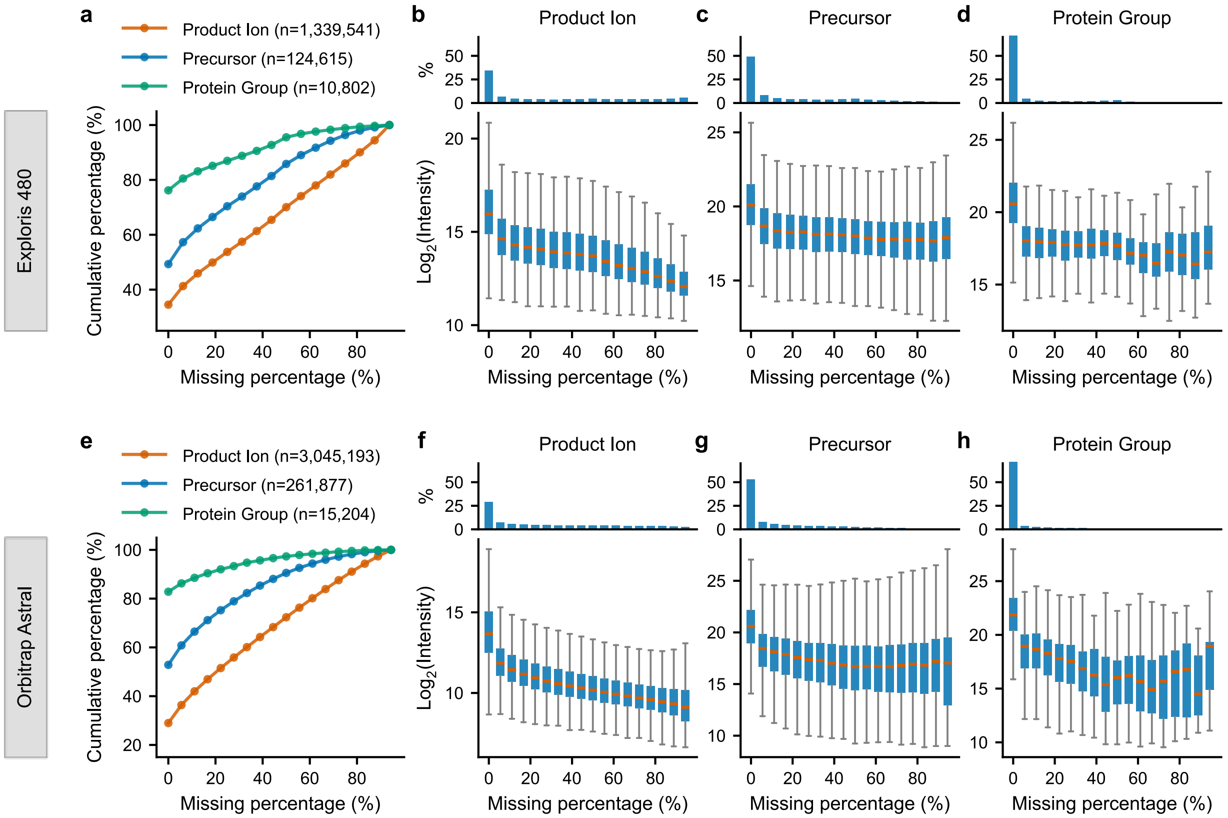

Supplementary Fig. 1. The extent of missingness at different quantification levels in Exploris 480 and Orbitrap Astral benchmark datasets. a–d, Exploris 480 dataset. **a**, Cumulative percentages of product ions, precursors, and protein groups on different levels of missingness. **b–d**, Relationship between missing-value percentage and $\log_2$ intensity at the product-ion (**b**), precursor (**c**), and protein-group (**d**) levels. **e–h**, Same as **a–d** for the Orbitrap Astral dataset.

bioRxiv preprint doi: https://doi.org/10.64898/2026.04.28.720212; this version posted May 1, 2026. The copyright holder for this preprint (which was not certified by peer review) is the author/funder, who has granted bioRxiv a license to display the preprint in perpetuity. It is made available under aCC-BY 4.0 International license.

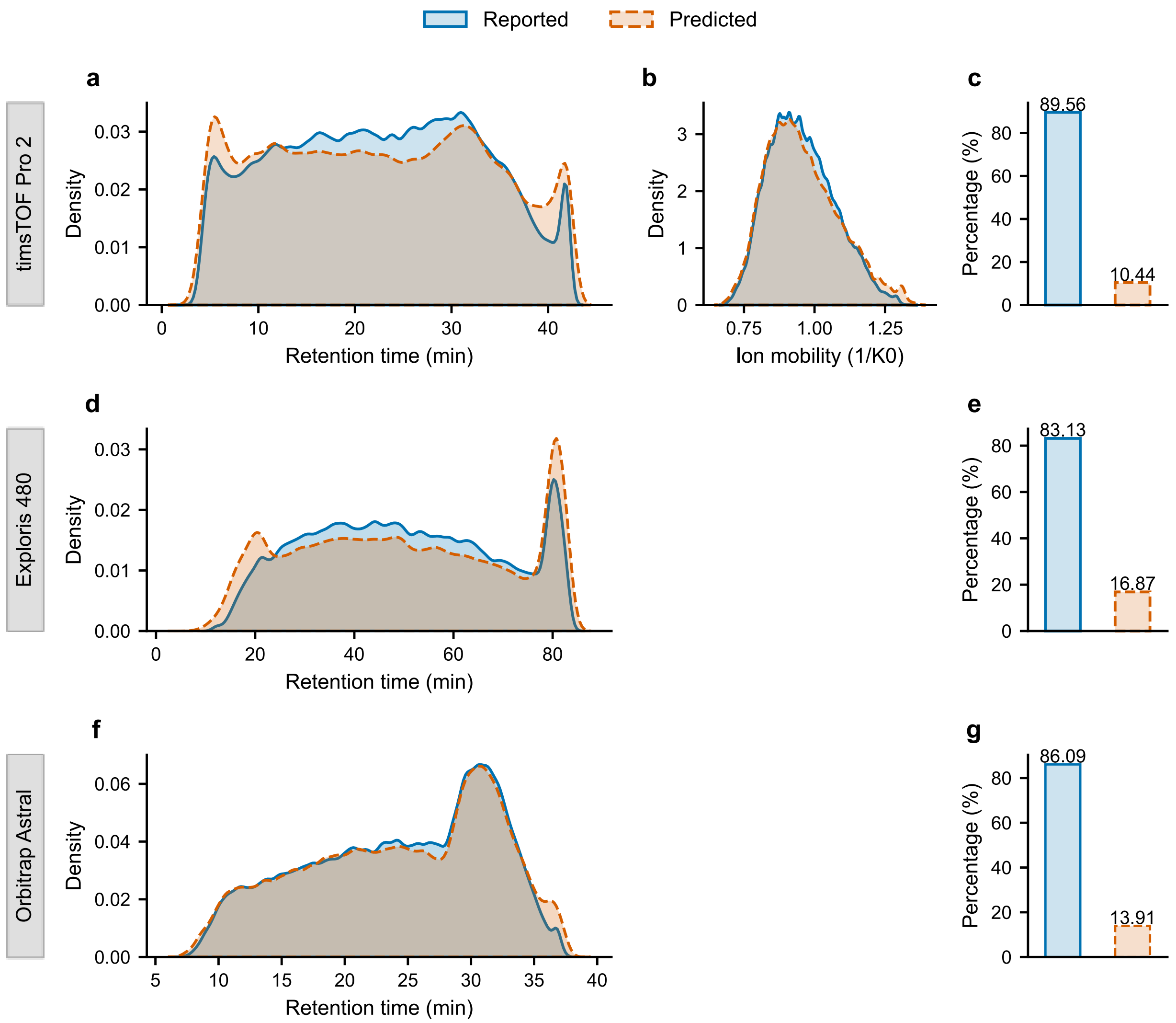

Supplementary Fig. 2. Reported and inferred chromatographic coordinates used for signal extraction. a–c, timsTOF Pro 2 dataset. **a**, Distributions of reported and JUMPlion-predicted retention times (RTs). **b**, Distributions of reported and JUMPlion-predicted ion mobility (IM) values. **c**, Proportions of reported and JUMPlion-predicted RT and IM values. **d,e**, Same as **a** and **c**, respectively, for the Exploris 480 dataset. **f,g**, Same as **a** and **c**, respectively, for the Orbitrap Astral dataset.

bioRxiv preprint doi: https://doi.org/10.64898/2026.04.28.720212; this version posted May 1, 2026. The copyright holder for this preprint (which was not certified by peer review) is the author/funder, who has granted bioRxiv a license to display the preprint in perpetuity. It is made available under aCC-BY 4.0 International license.

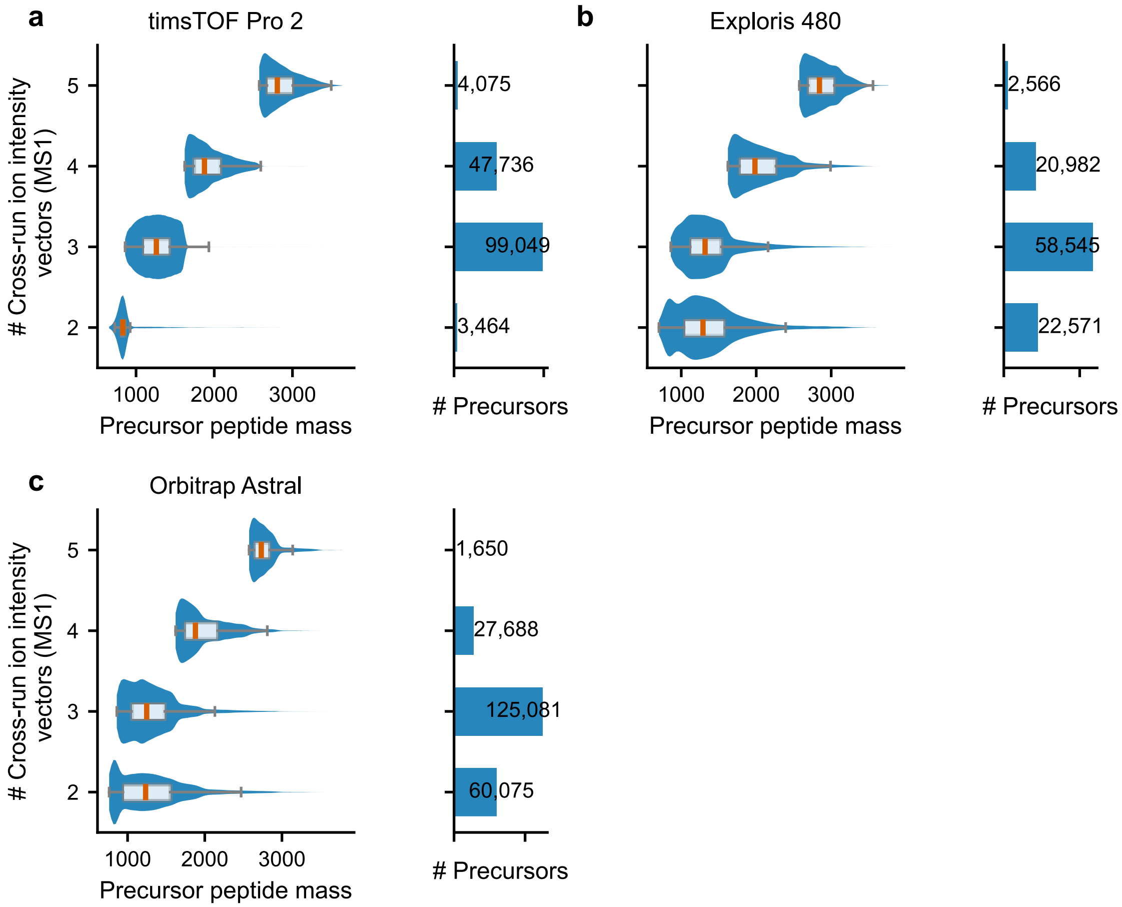

Supplementary Fig. 3. Relationship between precursor peptide mass and the number of MS1-level cross-run ion intensity vectors. a, timsTOF Pro 2 dataset. **b**, Exploris 480 dataset. **c**, Orbitrap Astral dataset.

bioRxiv preprint doi: https://doi.org/10.64898/2026.04.28.720212; this version posted May 1, 2026. The copyright holder for this preprint (which was not certified by peer review) is the author/funder, who has granted bioRxiv a license to display the preprint in perpetuity. It is made available under aCC-BY 4.0 International license.

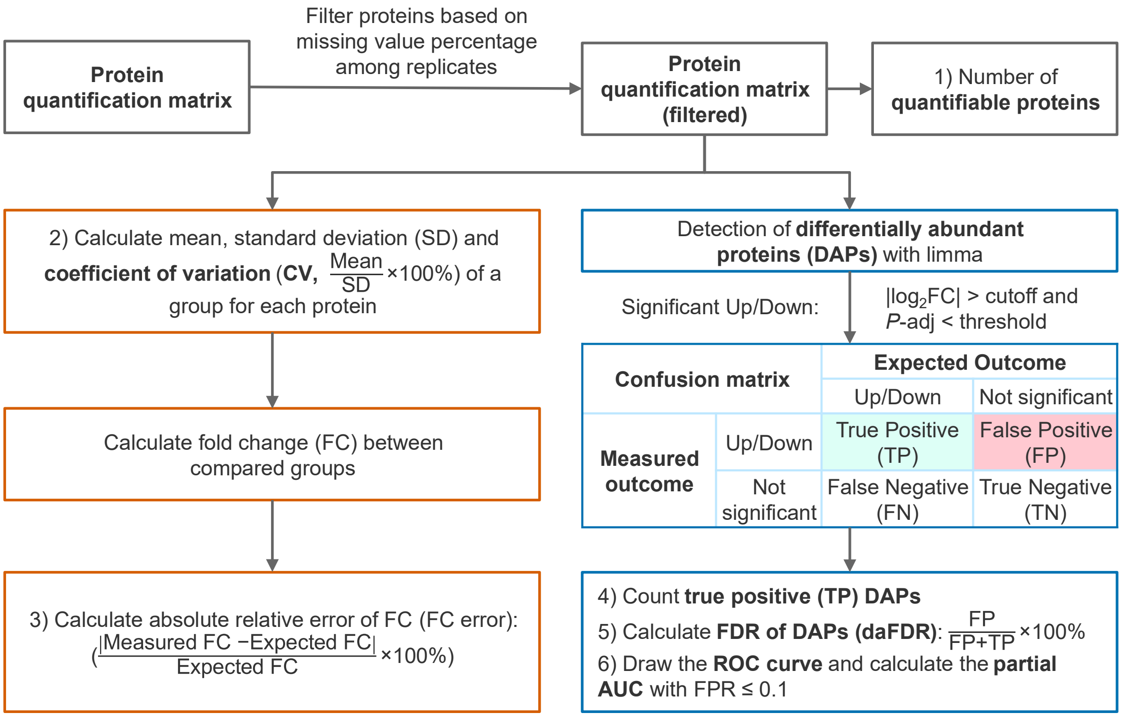

683

684 **Supplementary Fig. 4. The metrics used for evaluating the quantification performance.**

685

bioRxiv preprint doi: https://doi.org/10.64898/2026.04.28.720212; this version posted May 1, 2026. The copyright holder for this preprint (which was not certified by peer review) is the author/funder, who has granted bioRxiv a license to display the preprint in perpetuity. It is made available under aCC-BY 4.0 International license.

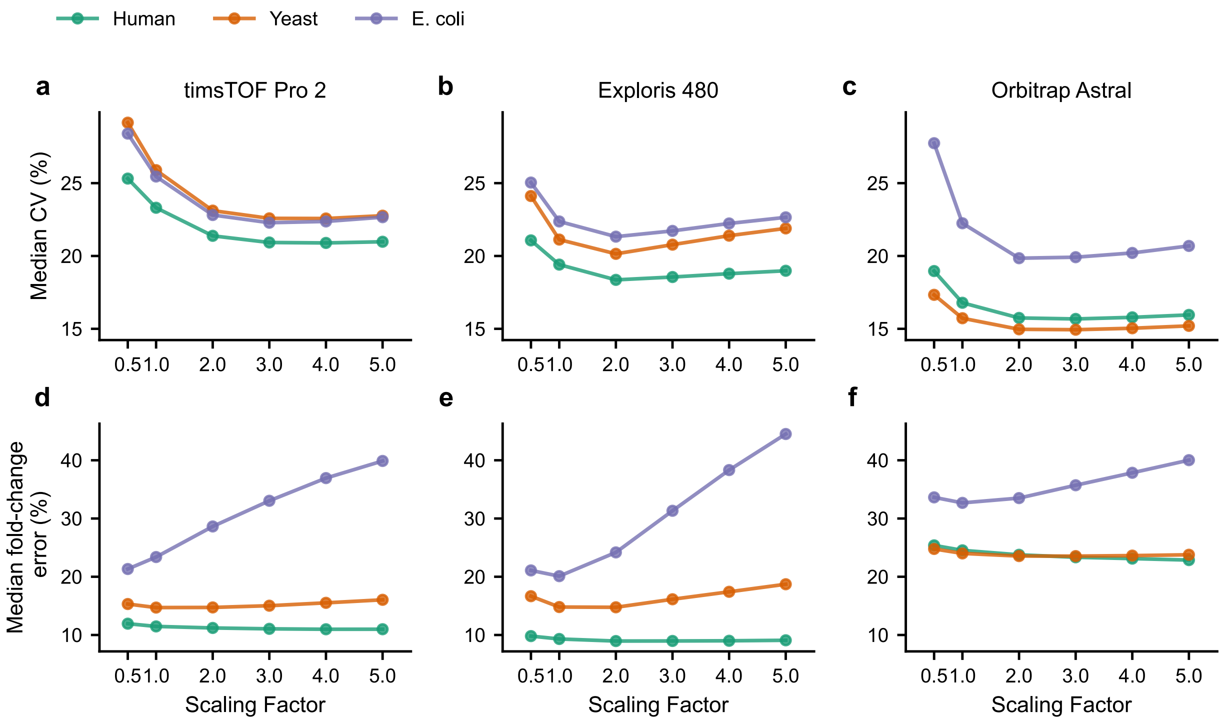

Supplementary Fig. 5. Effect of the local-minimum scaling factor on precursor-level quantification. a–c, Median coefficient of variation (CV) as a function of the scaling factor applied to the local minimum intensity for the timsTOF Pro 2 (**a**), Exploris 480 (**b**), and Orbitrap Astral (**c**) datasets. **d–f**, Median fold-change error for the corresponding datasets.

bioRxiv preprint doi: https://doi.org/10.64898/2026.04.28.720212; this version posted May 1, 2026. The copyright holder for this preprint (which was not certified by peer review) is the author/funder, who has granted bioRxiv a license to display the preprint in perpetuity. It is made available under aCC-BY 4.0 International license.

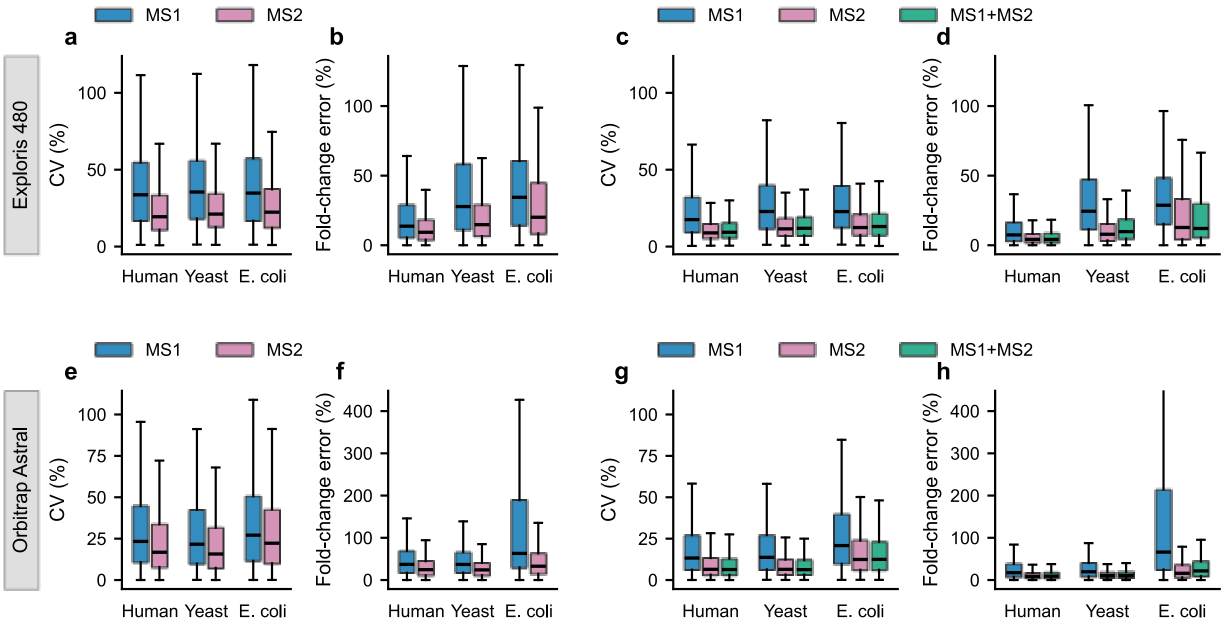

Supplementary Fig. 6. Precursor- and protein-level quantification performance in additional DIA platforms. a–d, Exploris 480 dataset. **a,b**, CV (**a**) and fold-change error (**b**) of precursor quantities summarized using LION-inferred MS1- and MS2-level ion intensities. **c,d**, CV (**c**) and fold-change error (**d**) of protein quantities summarized using MS1-only, MS2-only, and integrated MS1/MS2 strategies. **e–h**, Same as **a–d** for the Orbitrap Astral dataset.

bioRxiv preprint doi: https://doi.org/10.64898/2026.04.28.720212; this version posted May 1, 2026. The copyright holder for this preprint (which was not certified by peer review) is the author/funder, who has granted bioRxiv a license to display the preprint in perpetuity. It is made available under aCC-BY 4.0 International license.

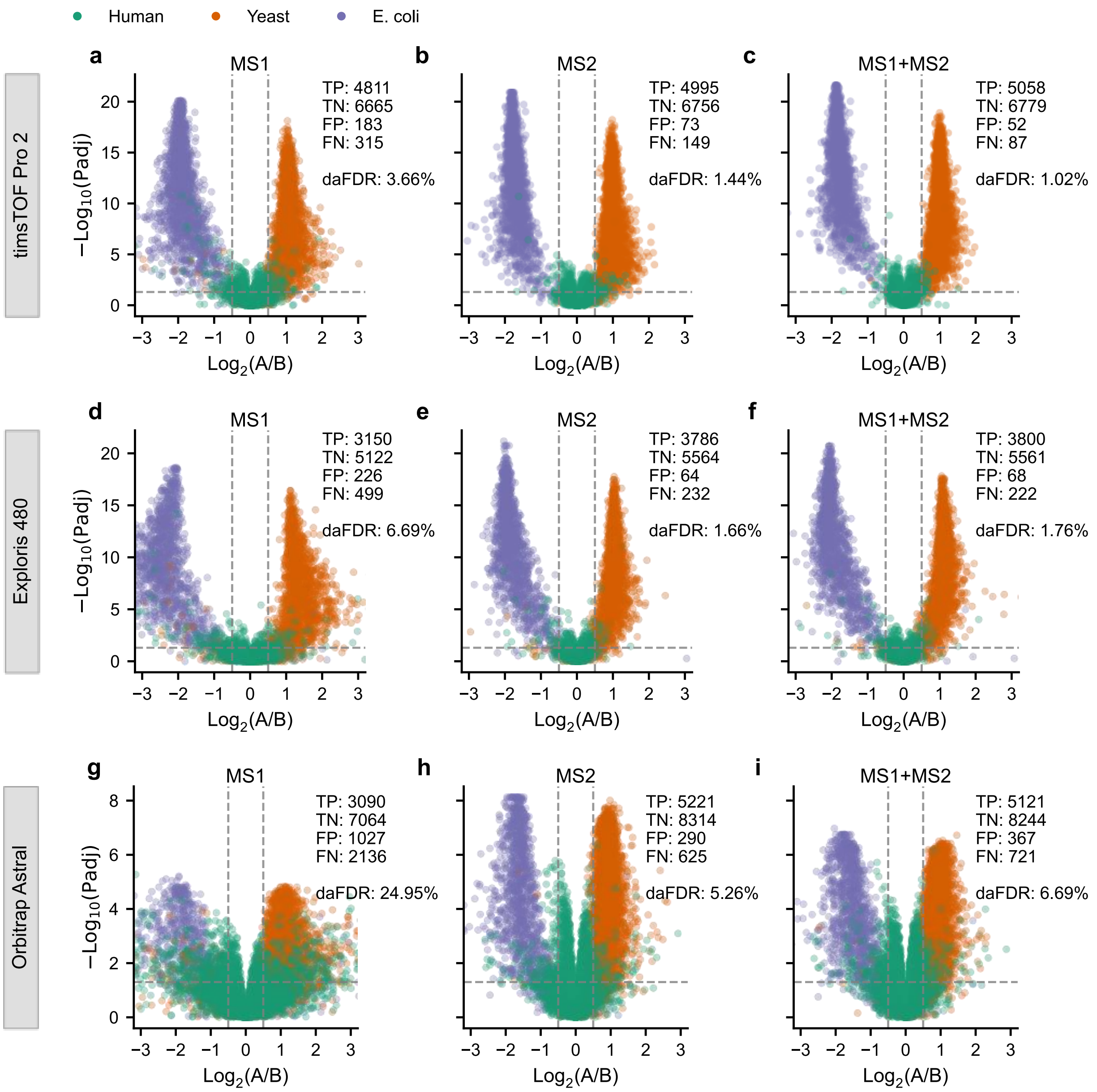

Supplementary Fig. 7. Differential abundance analyses for the protein quantities summarized using MS1-only, MS2-only and integrated MS1/MS2 approaches. a–c, timsTOF Pro 2 dataset. Volcano plots of differential protein abundance analysis using protein quantities summarized using MS1-only (**a**), MS2-only (**b**) and integrated MS1/MS2 (**c**) approaches. Points are colored by protein species of origin. The numbers of true positives (TP), true negatives (TN), false positives (FP) and false negatives (FN), along with the differential abundance false discovery rate (daFDR; FP/(TP+FP)), are indicated. **d–f**, same as **a–c** but for the Exploris 480 dataset. **g–i**, same as **a–c** but for the Orbitrap Astral dataset.

bioRxiv preprint doi: https://doi.org/10.64898/2026.04.28.720212; this version posted May 1, 2026. The copyright holder for this preprint (which was not certified by peer review) is the author/funder, who has granted bioRxiv a license to display the preprint in perpetuity. It is made available under aCC-BY 4.0 International license.

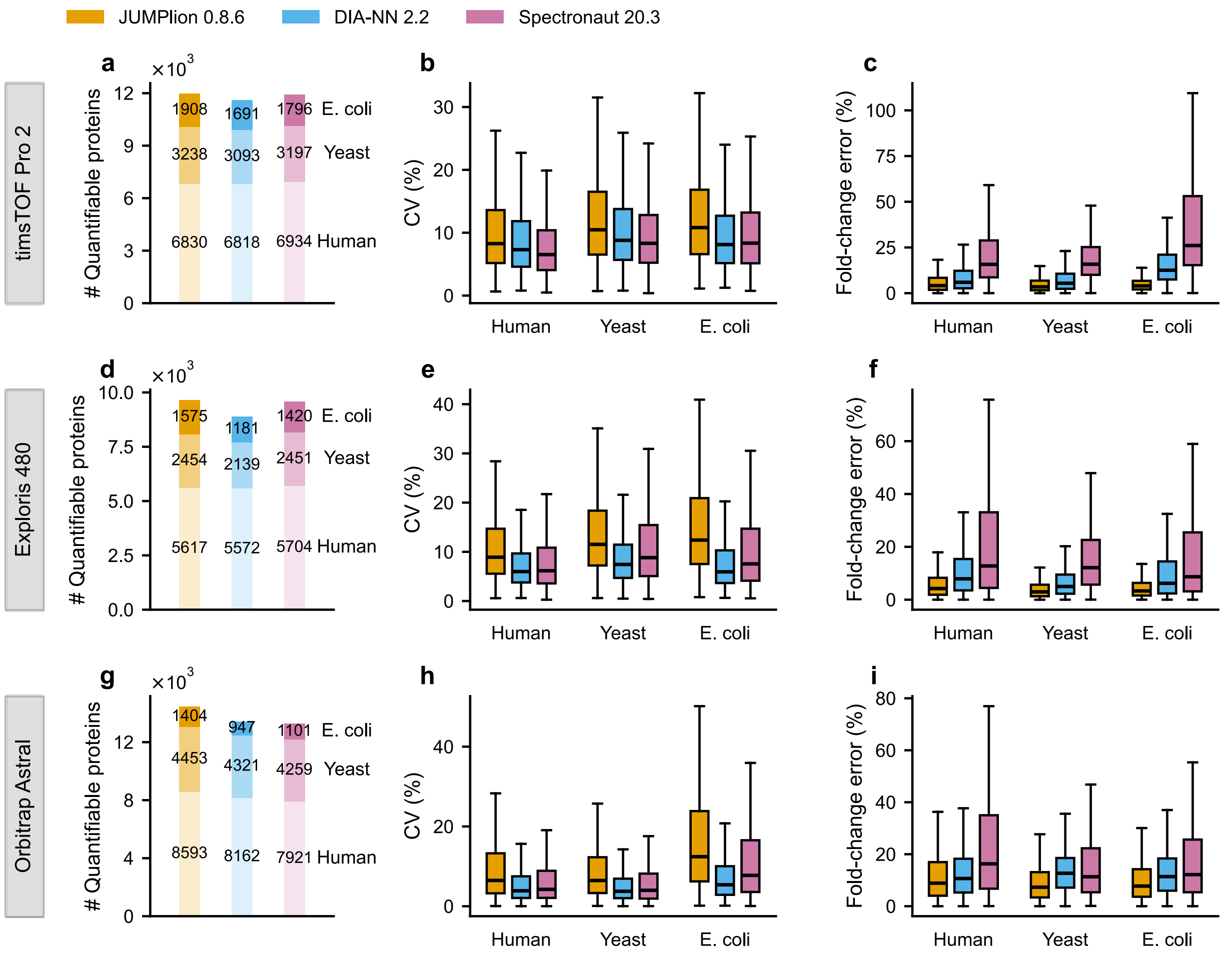

Supplementary Fig. 8. Protein-level quantification performance in JUMPlion v0.8.6, DIA-NN v2.2 and Spectronaut v20.3. a–c, timsTOF Pro 2 dataset. Number of quantifiable proteins (**a**), CV (**b**) and fold-change error (**c**) based on protein quantities derived from JUMPlion v0.8.6, DIA-NN v2.2 and Spectronaut v20.3. **d–f**, same as **a–c** but for the Exploris 480 dataset. **g–i**, same as **a–c** but for the Orbitrap Astral dataset.

bioRxiv preprint doi: https://doi.org/10.64898/2026.04.28.720212; this version posted May 1, 2026. The copyright holder for this preprint (which was not certified by peer review) is the author/funder, who has granted bioRxiv a license to display the preprint in perpetuity. It is made available under aCC-BY 4.0 International license.

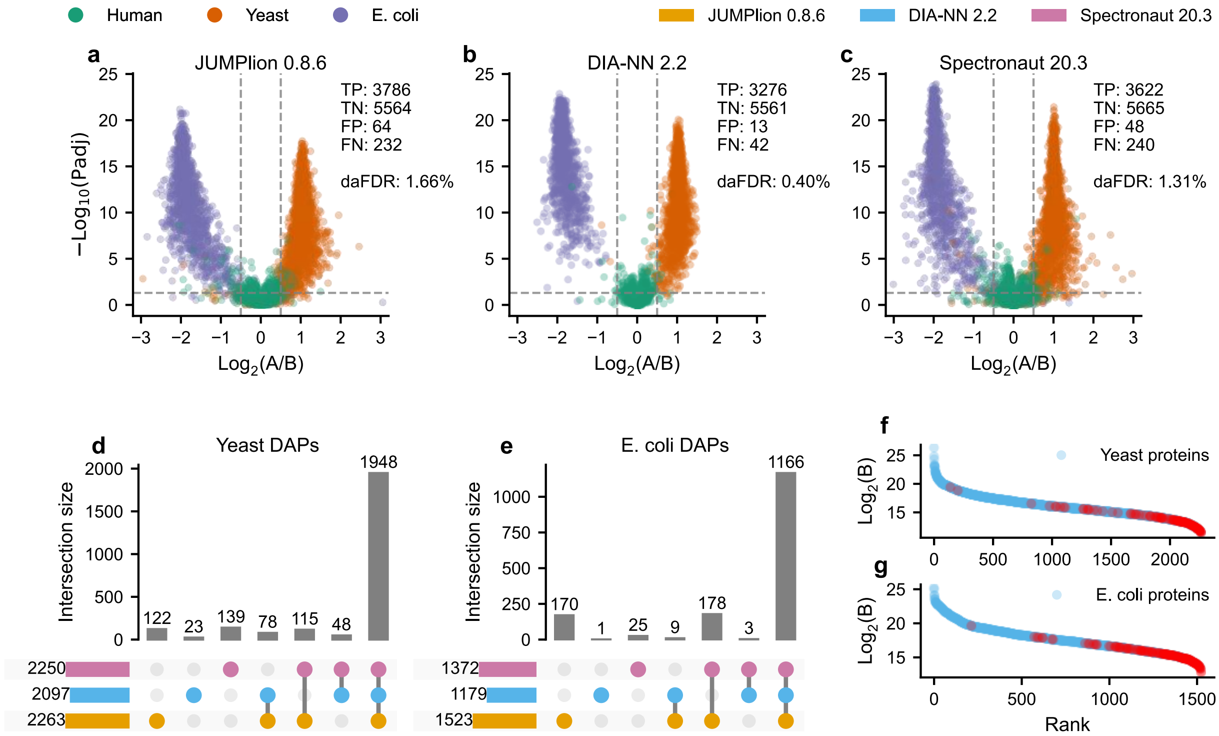

Supplementary Fig. 9. Differential abundance analyses for the Exploris 480 dataset. a–c, Volcano plots of differential protein abundance analysis using protein quantities derived from JUMPlion v0.8.6 (**a**), DIA-NN v2.2 (**b**) and Spectronaut v20.3 (**c**). Points are colored by protein species of origin. The numbers of TP, TN, FP and FN, along with the daFDR (FP/(TP+FP)), are indicated. **d,e**, UpSet plots showing unique and shared differentially abundant proteins (DAPs) from yeast (d) and *E. coli* (e) identified by JUMPlion, DIA-NN and Spectronaut. **f,g**, Ranked $\log_2$ intensities of all yeast and *E. coli* proteins, with DAPs uniquely identified using JUMPlion-derived quantities highlighted in red.

bioRxiv preprint doi: https://doi.org/10.64898/2026.04.28.720212; this version posted May 1, 2026. The copyright holder for this preprint (which was not certified by peer review) is the author/funder, who has granted bioRxiv a license to display the preprint in perpetuity. It is made available under aCC-BY 4.0 International license.

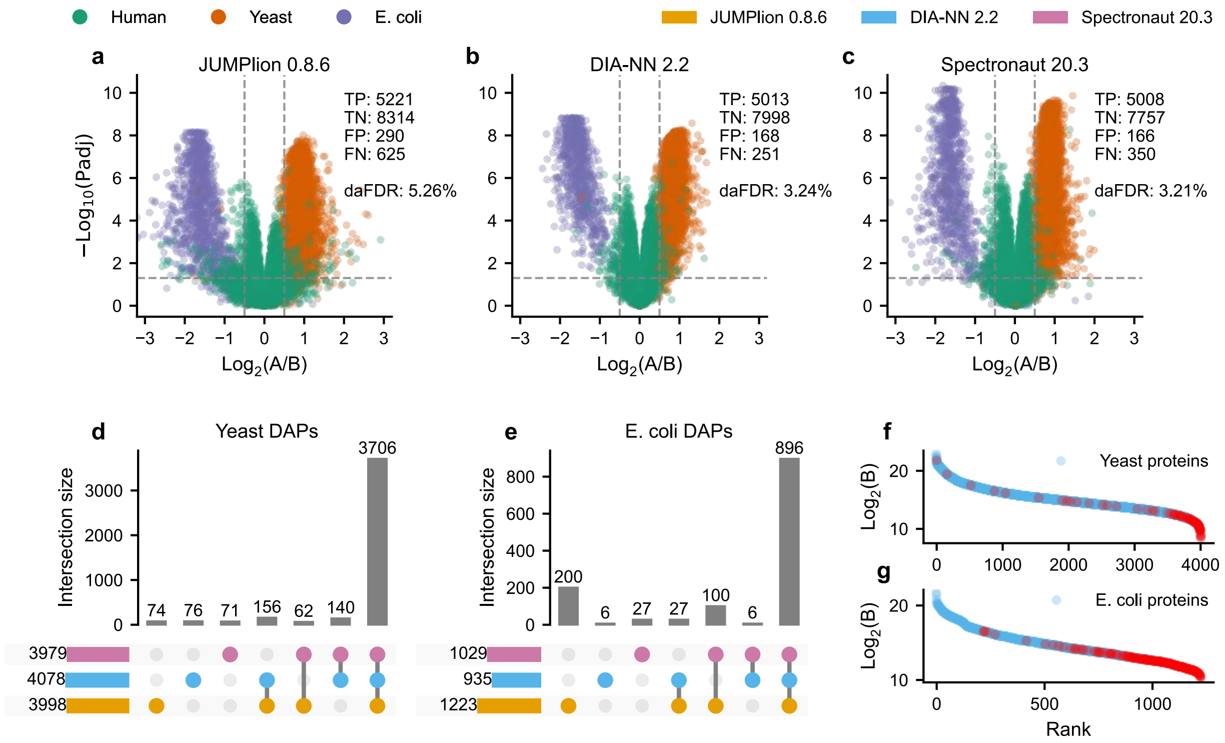

Supplementary Fig. 10. Differential abundance analyses for the Orbitrap Astral dataset. a–c, Volcano plots of differential protein abundance analysis using protein quantities derived from JUMPlion v0.8.6 (a), DIA-NN v2.2 (b) and Spectronaut v20.3 (c). Points are colored by protein species of origin. The numbers of TP, TN, FP and FN, along with the daFDR (FP/(TP+FP)), are indicated. **d,e**, UpSet plots showing unique and shared DAPs from yeast (d) and *E. coli* (e) identified by JUMPlion, DIA-NN and Spectronaut. **f,g**, Ranked $\log_2$ intensities of all yeast and *E. coli* proteins, with DAPs uniquely identified using JUMPlion-derived quantities highlighted in red.

bioRxiv preprint doi: https://doi.org/10.64898/2026.04.28.720212; this version posted May 1, 2026. The copyright holder for this preprint (which was not certified by peer review) is the author/funder, who has granted bioRxiv a license to display the preprint in perpetuity. It is made available under aCC-BY 4.0 International license.

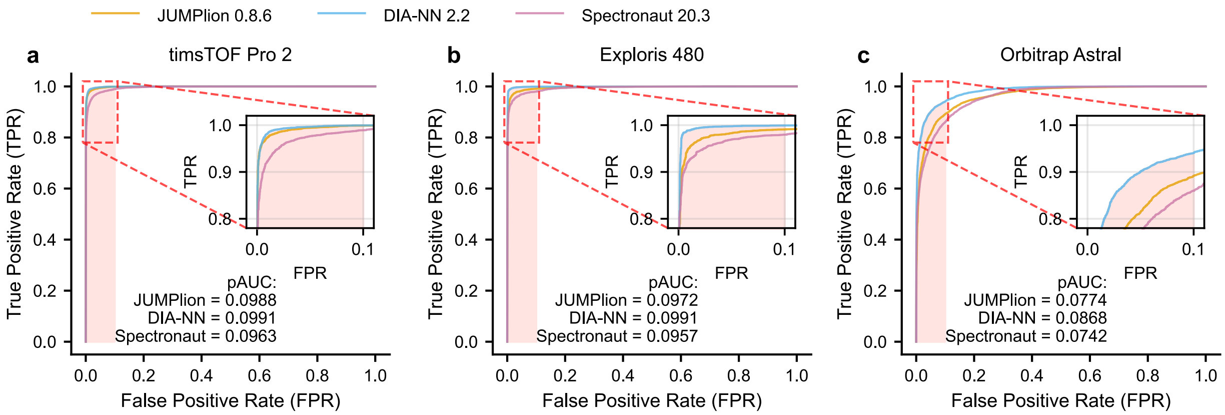

Supplementary Fig. 11. Receiver operating characteristic (ROC) curves for differential abundance benchmarking. ROC curves for timsTOF Pro 2 (**a**), Exploris 480 (**b**) and Orbitrap Astral (**c**) datasets were generated using $-\log_{10}$(adjusted P) as the ranking score. The partial area under the curve (pAUC) within false positive rate (FPR) ≤ 0.1 is highlighted in light red.

bioRxiv preprint doi: https://doi.org/10.64898/2026.04.28.720212; this version posted May 1, 2026. The copyright holder for this preprint (which was not certified by peer review) is the author/funder, who has granted bioRxiv a license to display the preprint in perpetuity. It is made available under aCC-BY 4.0 International license.

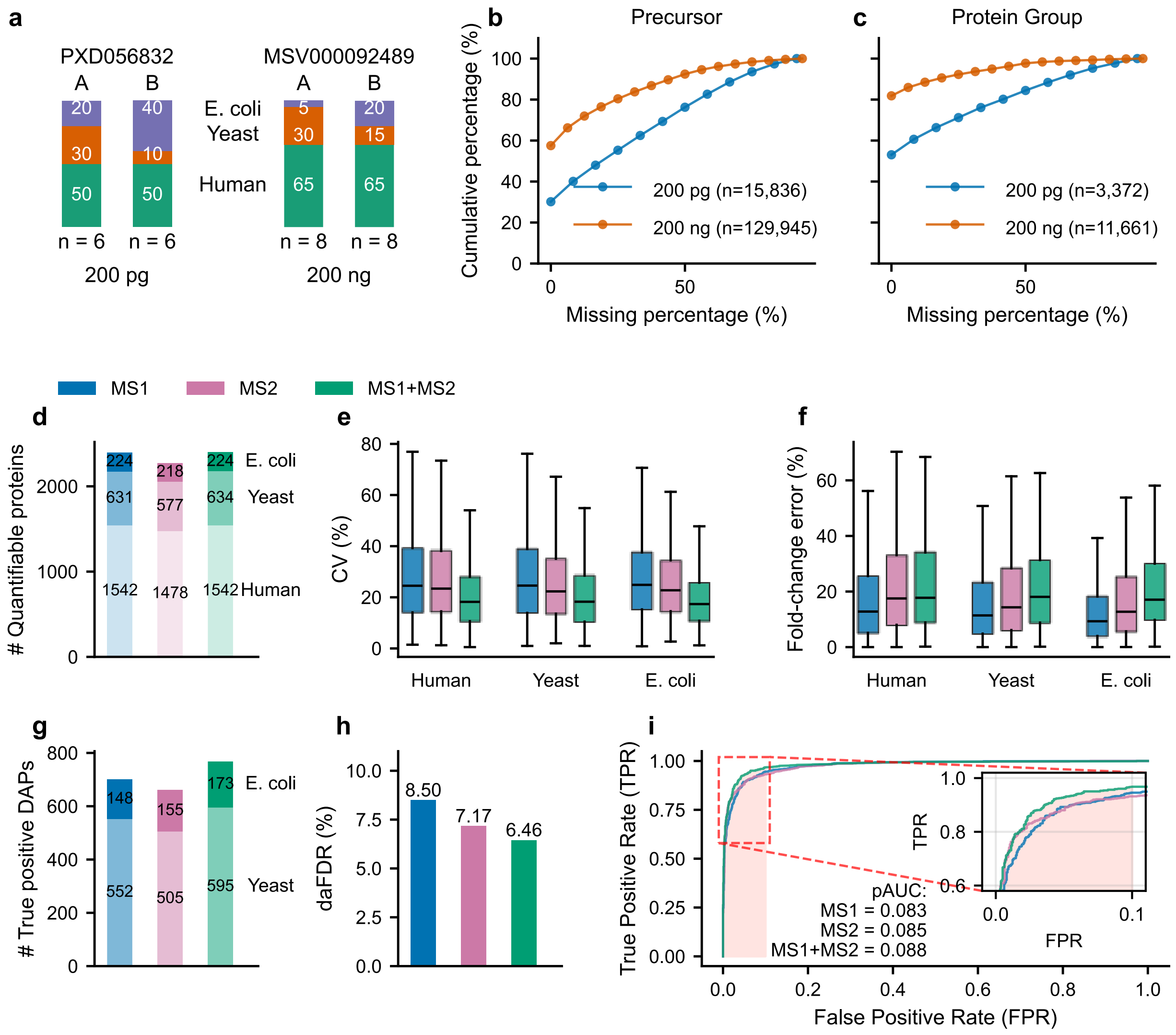

Supplementary Fig. 12. JUMPlion performance in low-input timsTOF Pro 2 DIA benchmarks. **a**, Overview of two timsTOF Pro 2 benchmark datasets differing by approximately three orders of magnitude in peptide loading amount (200 pg versus 200 ng). **b**,**c**, Cumulative percentages of precursors (**b**) and protein groups (**c**) on different levels of missingness in the 200 pg and 200 ng datasets. **d–i**, Quantification and differential abundance benchmarking for JUMPlion in the 200 pg dataset. **d**, Number of quantifiable proteins. **e**, CV. **f**, Fold-change error. **g**, Number of true positive DAPs. **h**, daFDR. **i**, ROC curves based on protein quantities summarized using MS1-only, MS2-only, and integrated MS1/MS2 strategies.

bioRxiv preprint doi: https://doi.org/10.64898/2026.04.28.720212; this version posted May 1, 2026. The copyright holder for this preprint (which was not certified by peer review) is the author/funder, who has granted bioRxiv a license to display the preprint in perpetuity. It is made available under aCC-BY 4.0 International license.

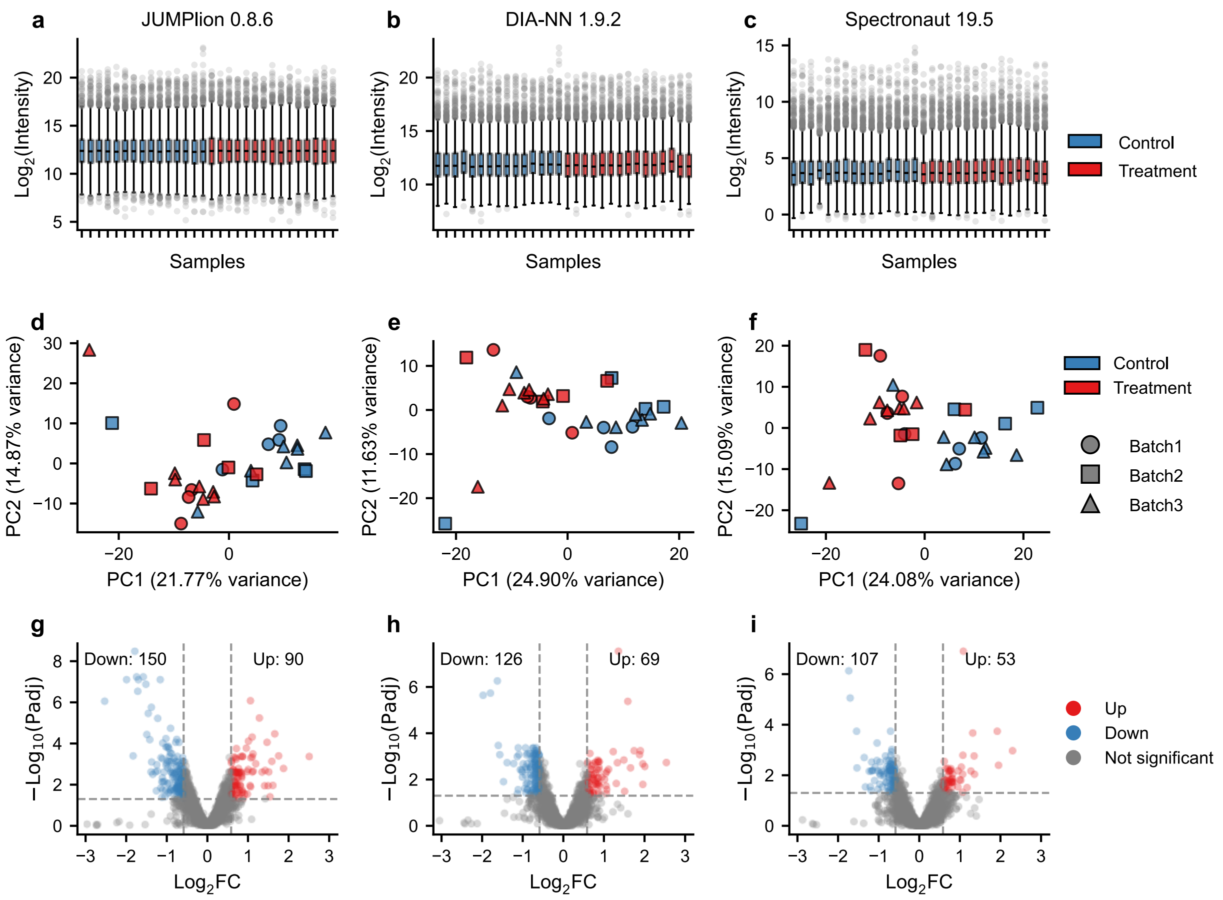

Supplementary Fig. 13. Normalization, batch correction, and differential abundance analysis of single-cell DIA data. a–c, Box plots of $\log_2$ protein intensities derived from JUMPlion (**a**), DIA-NN (**b**), and Spectronaut (**c**) after SCnorm normalization and limma-based batch correction. **d–f**, Principal component analysis plots generated using the top 500 most variable proteins in each quantification matrix. **g–i**, Volcano plots of differential protein abundance analysis using protein quantities derived from JUMPlion (**g**), DIA-NN (**h**), and Spectronaut (**i**). Points are colored as up-regulated, down-regulated, or not significant. The numbers of up- and down-regulated proteins are indicated.

bioRxiv preprint doi: https://doi.org/10.64898/2026.04.28.720212; this version posted May 1, 2026. The copyright holder for this preprint (which was not certified by peer review) is the author/funder, who has granted bioRxiv a license to display the preprint in perpetuity. It is made available under aCC-BY 4.0 International license.

Supplementary Table 1. Benchmark DIA-MS datasets used in cross-platform analyses.

Samples	Mass spectrometer	Loading amount	Number of runs	Database ID	Reference
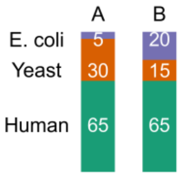	timsTOF Pro 2	400 ng	16<br>(n = 8 per sample)	MSV000092489	ref. 28
	Exploris 480	120 ng			
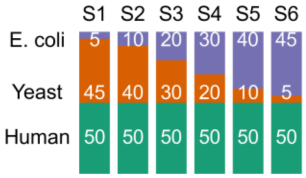	Orbitrap Astral	200 ng	18<br>(n = 3 per sample)	PXD046444	ref. 29

Samples from the S2 and S4 groups in the Orbitrap Astral dataset were used for differential abundance analysis to achieve expected fold changes for yeast and *E. coli* proteins similar to those in the timsTOF Pro 2 and Exploris 480 datasets.
